# Cryospray ablation (CSA) in the palliative treatment of squamous cell carcinoma of the esophagus

**DOI:** 10.1186/1477-7819-5-34

**Published:** 2007-03-16

**Authors:** Brooks D Cash, Lavonne R Johnston, Mark H Johnston

**Affiliations:** 1 Department of Gastroenterology, National Naval Medical Center, Bethesda, MD 8901 Wisconsin Avenue, Bldg 9, Department of Gastroenterology, Bethesda, MD 20889, USA; 2 Lancaster Gastroenterology, Inc., 2112 Harrisburg Pike, Suite 202, PO Box 3200, Lancaster, PA 17604-3200, USA

## Abstract

**Background:**

Esophageal carcinoma is the ninth most prevalent cancer worldwide with squamous cell carcinoma (SCCA) and adenocarcinoma accounting for the vast majority of new cases (13,900 in 2003). Cure rates in the U.S. are less than 10%, similar to lung cancer. More than 50% of patients with esophageal carcinoma present with unresectable or metastatic disease, are not surgical candidates, or display disease progression despite the addition of neoadjuvant chemoradiotherapy to surgery. Need for improved palliation exits.

**Case presentation:**

This case describes a 73-year-old African American male who presented with recurrent squamous cell carcinoma (SCCA) of the esophagus who has a achieved complete remission for 24 months via endoscopic cryospray ablation.

**Conclusion:**

Endoscopic cryo spray ablation warrants further investigation as a palliative treatment modality for esophageal cancer. This is the first reported case in the medical literature.

## Background

Esophageal carcinoma is the ninth most prevalent cancer worldwide with squamous cell carcinoma (SCCA) and adenocarcinoma accounting for the vast majority of new cases (13,900 in 2003 in the USA) [1,2]. Cure rates in the U.S. are less than 10%, similar to lung cancer [3]. The definitive surgical therapy, esophagectomy, can provide 10–26%, 5-year, disease-free, all-stage survival rates [3,4]. However, more than 50% of patients with esophageal carcinoma present with unresectable or metastatic disease and are not surgical candidates, or display disease progression despite the addition of neoadjuvant chemoradiotherapy to surgery [3-5].

Cryospray ablation (CSA) using liquid nitrogen sprayed through a low pressure device has recently been described to be effective and safe in the treatment of Barrett's esophagus including high-grade dysplasia [6-12]. This case report describes the first use of CSA in a patient with recurrent SCCA for palliative treatment that has resulted in complete remission for 2 years.

## Case presentation

**Figure 1:**
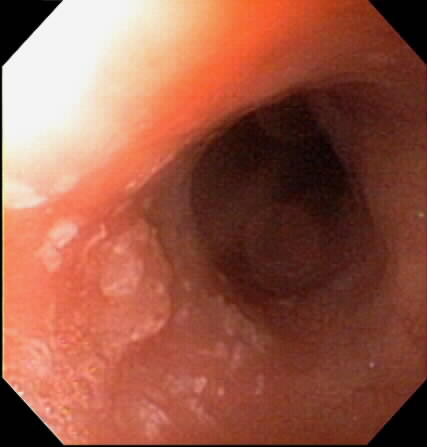
Squamous cell carcinoma (SCCA) in the proximal esophagus at 24 cm from the incisors. This lesion occurred above the prior radiation therapy treatment field.

**Figure 2:**
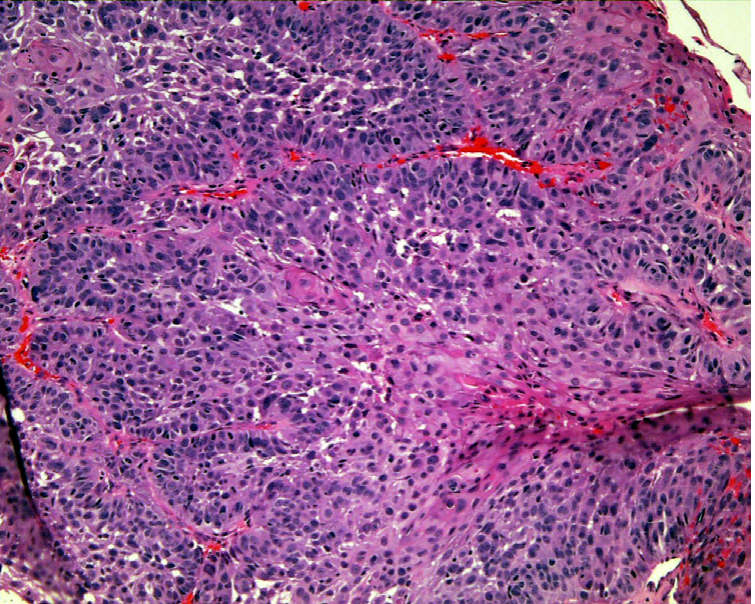
Histology of figure 1. This is a moderately differentiated SCCA (H&E).

A 73 year old African American male with history of SCCA of the esophagus presented with mild dysphagia. Subsequent endoscopy (EGD) revealed recurrent SCCA of the esophagus. Seven years prior he was diagnosed with a T1-2, N1 SCCA of the left anterior tonsillar pillar and treated with radiation therapy (XRT) (6660 cyG to the primary tumor). Four years later he developed Stage III esophageal SCCA (T4, N0, M0), by chest CT, located at 33 cm from the incisors that was 3 cm in length. This lesion was treated with 5400 cGy XRT and chemotherapy with curative intent. On the third (current) presentation, a moderately differentiated, T2, by endoscopic ultrasound (EUS), SCCA lesion at 24 cm was discovered (Figure 1 and 2). This new lesion was above the prior field of XRT and proximal to the original stage III esophageal SCCA.

**Figure 3:**
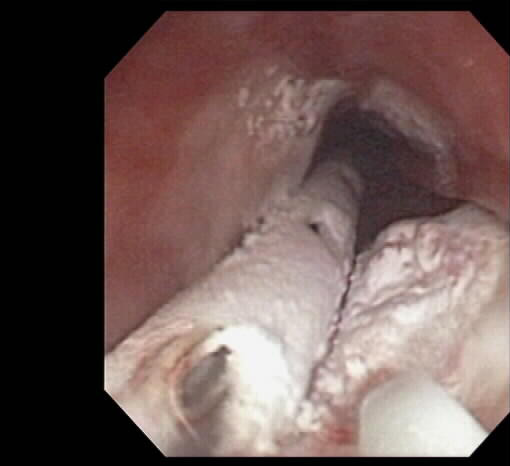
Cryospray ablation (CSA) of the SCCA in figure 1. On the left is the cryo decompression tube. In the right fore corner is the cryo catheter.

The patient was evaluated by the institution's tumor board and deemed neither a surgical nor XRT candidate based on significant cardiac comorbidities and maximum dose XRT with his prior treatments. Palliative treatment options were explored. Based on the success with ablation of esophageal lesions as described above, palliation via CSA was pursued for this patient. At the time of this case, the device was under an investigational device exemption (IDE) issued by the FDA. Therefore, an FDA Humanitarian Use Device exemption was requested and granted for use of the CSA device. The patient signed a consent form approved by the authors IRB and the FDA. The patient's 2 cm long, hemi-circumferential SCCA was treated with two 30 second pulses of CSA under direct endoscopic visualization on an outpatient basis (Figure 3). This particular dosimetry was chosen based on early clinical trials in Barrett's esophagus and swine data. The first CSA of Barrett's esophagus in clinical trials used a dose of 40 seconds (two 20 second applications separated by a 20–30 second thaw). Early animal studies at the author's institution (unpublished) have demonstrated that the depth of injury correlates with duration of freeze. High grade dysplasia has been effectively treated with CSA at 20 seconds times three cycles. Thus in hope of achieving greater depth of injury without excessive necrosis, two cycles of 30 seconds each were applied. CSA was applied in such a way that the entire tumor with margins of 1–2 cm was frozen. This technique resulted in a near circumferential freeze of the esophagus but with a focus on the tumor which endoscopically appeared hemi-circumferential in distribution. Interestingly, freezing of the tumor under direct endoscopic visualization demonstrated that the tumor differentially retracted when frozen relative to the surrounding esophageal mucosa making the margins of the tumor more distinct. Prior to freezing, the margins of the tumor diffusely blended into the esophageal wall.

**Figure 4:**
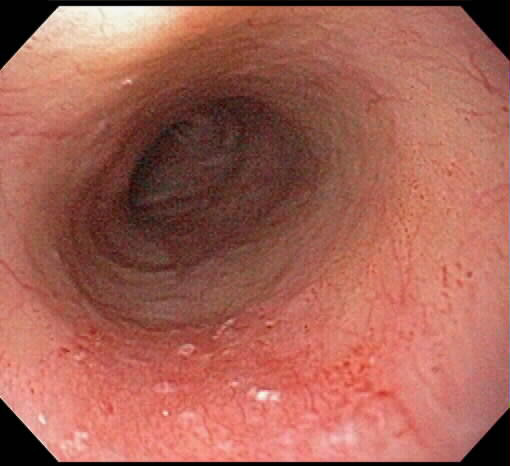
Endoscopic appearance of the SCCA (figure 1) one month after cryospray ablation.

This patient was treated with lansoprazole 30 mg BID throughout the CSA period. The patient experienced no initial complications. One month latter a follow-up EGD revealed endoscopic resolution of the tumor, but biopsies remained positive for moderately differentiated SCCA (Figure 4). CSA of the area was repeated, treating 40% of the esophageal circumference 4 cm in length spanning the prior SCCA with three, 20 second pulses. This shorter pulsed duration of CSA was chosen because less depth of injury was desired based on the significant response with the initial treatment. Increased numbers of CSA cycles are associated with greater tissue injury. Therefore the intent was a more intense treatment but with less depth of injury. Twenty-four hours later, the patient experienced odynophagia requiring oral narcotics for 3 weeks followed by the development of a stricture at one month post-CSA. This was treated with Savary dilation but has subsequently become a persistent esophageal stricture at that the site. Biopsies at the cancer site 6 weeks after the second cryoablation and bimonthly in the subsequent 12 months of follow-up were completely negative for dysplasia or neoplasia.

**Figure 5:**
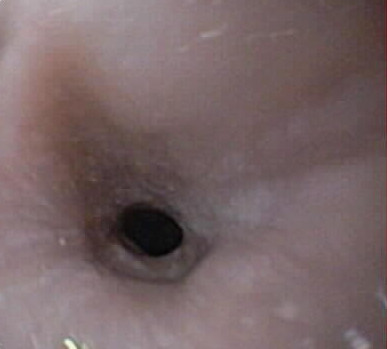
Fibrotic stricture which developed after the second cryospray ablation treatment.

The persistent esophageal stricture (Figure 5) has required multiple dilations with various types of dilators (with and without corticosteroid injections) and temporary (3 month) esophageal stent (Polyflex^® ^by Wilson Cook) placement. The patient continues to work and remains cancer free 24 months post-treatment.

## Discussion

Despite ongoing research in esophageal cancer, the overall median survival following curative esophagectomy has been shown to be 34–38.6 months [13,14] with a 7 month median survival following detection of recurrent esophageal cancer [14]. Palliation therapies are needed for both survival prolongation and symptom relief. Current palliation approaches include feeding tubes, dilation, stents, radiation, chemotherapy and various ablative techniques, all of which have benefits and risks [4,15].

The mechanism of action underlying the injury caused by CSA is unique for an ablative technique. CSA induces apoptosis and causes cryonecrosis at super-cold temperatures (-76°C to -158°C), which results in transient ischemia at the CSA site and can cause immune stimulation [16]. Some precancerous lesions such as Barrett's epithelium and many cancers are resistant to apoptosis and therefore might be uniquely suited for treatment by CSA [17]. Work in the swine has laid the foundations for the use of CSA. Initially, research was conducted to define the optimum cryogen dosimetry. This was followed by depth-of-injury work using endoscopic ultrasonography (EUS) and then comparative trials of CSA, MPEC and APC. Depth of injury at doses of 20 seconds times two cycles approximates 2 mm. Longer duration freezes achieve greater depths of injury [11].

It is unclear at this point what stages of esophageal cancer could potentially be treated/palliated with this modality. However, in this particular case and based on the degree of stricturing that followed it is likely that full esophageal wall thickness injury occurred. Thus palliation of tumors up to T4 (tumor invading adjacent structures) is conceivable.

The stricture that developed in this case was significant and required multiple interventions. Other potential complications of CSA include perforation, and bleeding. It is unknown what the actual complication rates are for this modality at this dose due to its relative novelty. In the first trial published by Johnston *et al*., no complications were reported. They used a dosimetry of 20 seconds times two for the first 10 patients. The CSA complication rate relative to other ablation techniques such as photodynamic therapy (PDT) is also unknown. However, for PDT the esophageal stricture rate is 30 to 50% depending on the number of treatments.

This particular malignancy may have been a very indolent one with slow growth as evidenced by the significant time interval between the first and second esophageal cancer presentation. It is also conceivable that only one CSA treatment was necessary for the palliation of this tumor in that "cure" is highly unlikely in such a case. One application of CSA may have avoided the subsequent stricture. However, this case still represents the first of its kind in the medical literature using this device and technique to achieve a sustained remission for SCCA of the esophagus. CSA has advantages relative to other palliative measures in that it is simple to use, can be performed with a standard diagnostic upper endoscope, is relatively inexpensive, and has a low rate of complications [12]. There is no photosensitivity as in PDT and most patients after CSA can resume eating and a normal lifestyle the same day. Although treatment of this patient resulted in a post treatment stricture, he has no evidence of recurrent malignancy two years post treatment. A sustained remission of this nature in recurrent SCCA is unusual. Although speculative, this sustained remission could relate to the unique mechanisms of action of CSA. These mechanisms of injury include the induction of apoptosis, cryonecrosis, and systemic anti-tumor immune stimulation [18-20]. The effectiveness of CSA in the treatment of neoplasms such as prostate cancer can also be enhanced by certain chemotherapeutic agents such as 5-fluorouracil [20]. These mechanisms of injury in concert with the method of delivery may make CSA an ideal option for the palliative treatment of esophageal neoplasms. Pilot studies indicate that mucosa damaged by cryotherapy followed by healing in an acid-free environment results in re-epithelialization with normal squamous epithelium in the majority of patients [12].

## Conclusion

Some precancerous lesions such as Barrett's epithelium and many cancers are resistant to apoptosis and therefore might be uniquely suited for treatment by CSA. This case report describes the first use of CSA in a patient with recurrent SCCA for palliative treatment that has resulted in complete remission for 2 years. Further investigation using CSA as a palliative measure for esophageal cancer should be considered.

## Competing interests

Dr. Brooks Cash: declares no competing interests

Dr. Mark Johnston: invented the CSA devices but has permanently divested himself of all financial interests and potential future royalties to an U.S.A. Internal Revenue Service approved 501c3 charity. Dr. Johnston has served as a consultant to CSA Medical Inc. the developer of the cryospray ablation device.

Lavonne Johnston, PA-C has been salaried as a research assistant funded through CSA Medical Inc.

## Authors' contributions

**BC **contributed to the editing of this manuscript and participated in the care of the case described.

**MJ **contributed to the editing of this manuscript and participated in the care of the case described.

**LJ**, PA-C contributed to the writing and editing of the submitted manuscript and has been the primary research assistant for this cryotherapy research.
